# Genetically predicted associations between inflammatory cytokines and juvenile idiopathic arthritis: A Mendelian randomization study

**DOI:** 10.1097/MD.0000000000051005

**Published:** 2026-10-02

**Authors:** Jian Pang, Lijia Pan, Pingping Liu, Wanyue Qin, Jinhua Yang, Yifei Feng, Sheng He, Shuihua Yang

**Affiliations:** a Department of Medical Ultrasonics, Maternal and Child Health Care Hospital of Guangxi Zhuang Autonomous Region, Nanning, Guangxi, China; b Guangxi Clinical Research Center for Birth Defects, Guangxi Key Laboratory of Reproductive Health and Birth Defects Prevention, Maternal and Child Health Hospital of Guangxi Zhuang Autonomous Region, Nanning, China.

**Keywords:** genetically predicted association, inflammatory cytokines, juvenile idiopathic arthritis, Mendelian randomization, single-nucleotide polymorphism

## Abstract

This study evaluates associations between genetically predicted circulating levels of 91 inflammatory cytokines and juvenile idiopathic arthritis (JIA) susceptibility. Summary statistics for 91 circulating inflammatory cytokines were obtained from a meta-analysis of 11 cohorts comprising 14,824 participants of European ancestry. JIA summary statistics were obtained from FinnGen Release 9 and included 788 cases and 172,834 controls of European ancestry. The primary estimator was the inverse-variance weighted (IVW) random-effects model. MR-Egger, weighted median, simple mode, and weighted mode analyses were used as complementary estimators. Sensitivity analyses included the MR-Egger intercept, Mendelian Randomization Pleiotropy RESidual Sum and Outlier, Cochran’s *Q*, MR-Steiger, and leave-one-out tests. The C–C motif chemokine ligand 19 (CCL19) analysis used 19 independent single-nucleotide polymorphism instruments. At the nominal threshold of *P* < .05, IVW estimates indicated associations of CCL19 (odds ratio [OR], 2.48), fibroblast growth factor 19 (OR, 0.78), and monocyte chemotactic protein 3 (OR, 0.79) with JIA. After Bonferroni correction for 91 tests (threshold *P* < 5.49 × 10^−4^), only CCL19 remained significant (IVW *P* = 8.93 × 10^−8^; adjusted *P* = 8.12 × 10^−6^). The direction of the CCL19 estimate was consistent across Mendelian randomization methods, although significant heterogeneity and attenuation after outlier correction using Mendelian Randomization Pleiotropy RESidual Sum and Outlier indicated uncertainty in its magnitude. Higher genetically predicted CCL19 levels were associated with greater JIA susceptibility in participants of European ancestry. These findings reflect genetically predicted associations rather than established causal effects and do not support clinical prediction or intervention. Independent genetic, experimental, and clinical validation is required before CCL19 can be considered a biomarker or therapeutic target.

## 1. Introduction

Juvenile idiopathic arthritis (JIA) denotes otherwise unexplained arthritis lasting more than 6 weeks in children and includes clinically distinct International League of Associations for Rheumatology categories.^[1]^ Persistent inflammation can cause disability, visual impairment, skeletal damage, and substantial healthcare use; only a proportion of affected children achieve sustained disease control during long-term follow-up.^[2,3]^

JIA pathogenesis is multifactorial and involves genetic susceptibility, environmental exposures, and immune dysregulation.^[4,5]^ Its phenotypic heterogeneity also implies that inflammatory pathways may differ across systemic, oligoarticular, polyarticular, enthesitis-related, and other disease categories.

Inflammatory cytokines regulate systemic inflammation, synovial injury, and bone remodeling in JIA.^[6]^ Imbalances among T-helper cell subsets and cytokine signaling involving interleukin-6 (IL-6), interleukin-17, tumor necrosis factor-alpha (TNF-α), and interferon-gamma are established features of JIA immunobiology.^[7–9]^

Because cytokines are pleiotropic, interdependent, and sensitive to disease activity and treatment, observational associations may reflect confounding or reverse causation.^[10,11]^ These features make it difficult to distinguish circulating markers of active inflammation from factors that contribute to disease susceptibility.

Genetic epidemiological approaches can complement clinical and experimental studies by testing whether inherited differences in cytokine levels are associated with JIA susceptibility while reducing, although not eliminating, conventional confounding and reverse causation.

Mendelian randomization (MR) uses genetic variants as instrumental variables (IVs) to estimate genetically predicted exposure–outcome associations.^[12–16]^ We therefore conducted a two-sample MR analysis of 91 circulating inflammatory cytokines and JIA. The study was intended to prioritize associations for replication and mechanistic investigation, not to establish clinical biomarkers or treatment targets.

## 2. Materials and methods

### 2.1. Study design

We conducted a unidirectional two-sample MR analysis using publicly available summary-level genome-wide association study (GWAS) data to evaluate associations between genetically predicted circulating inflammatory cytokine levels and JIA susceptibility. Heterogeneity, pleiotropy, directionality, and influence analyses were used to examine the robustness of the estimates. The contributing GWAS studies received approval from their respective ethics committees, and all participants provided informed consent. Because this secondary analysis used only deidentified, publicly available summary statistics and involved no new participant recruitment or individual-level data, no additional institutional review board approval or informed consent was required.

Valid MR inference depends on 3 core assumptions^[12]^ (Fig. 1): relevance – the selected IVs are associated with the cytokine exposure; independence – the IVs are independent of confounders of the exposure–outcome relationship; and exclusion restriction – the IVs influence JIA only through the cytokine exposure and not through alternative pathways.

**Figure 1.**
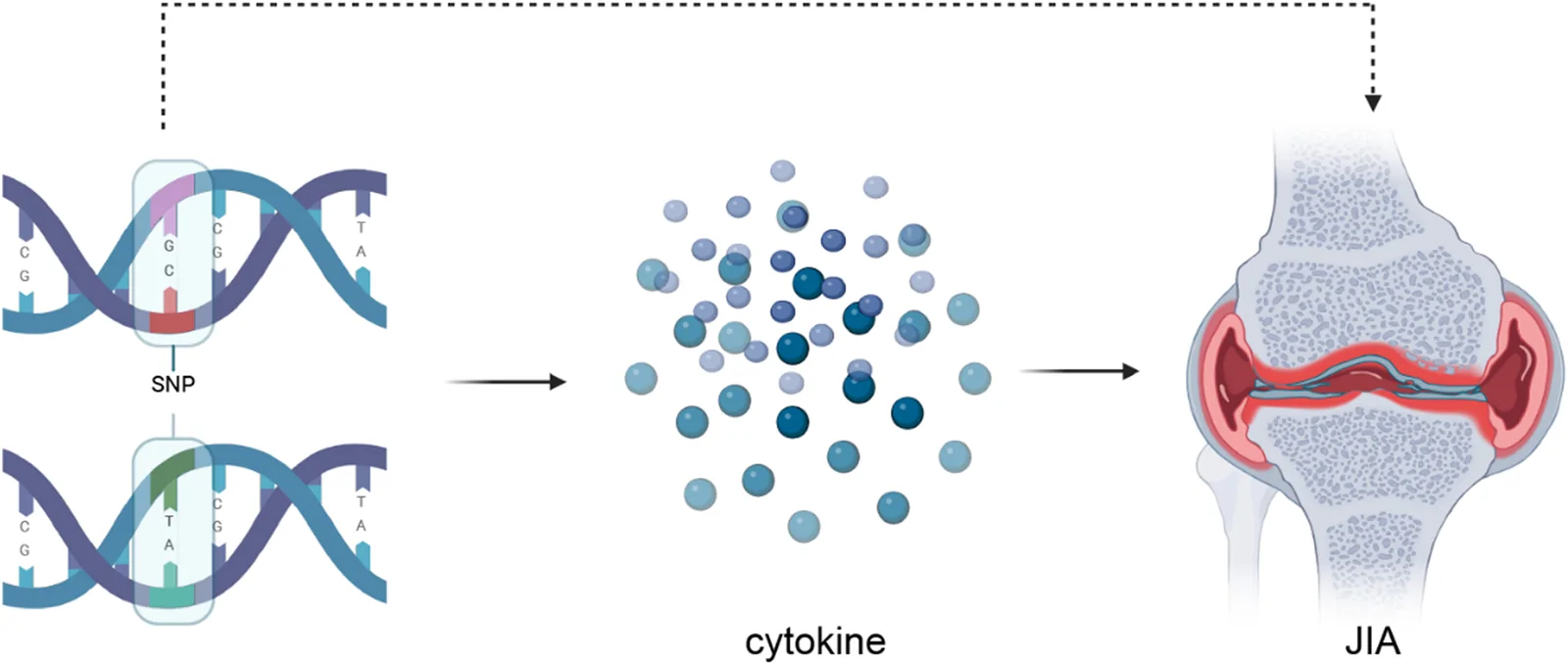
Conceptual framework for Mendelian randomization of circulating inflammatory cytokines and juvenile idiopathic arthritis. The design requires 3 assumptions: relevance (genetic variants are associated with cytokine levels), independence (variants are independent of exposure–outcome confounders), and exclusion restriction (variants affect JIA only through cytokine levels). JIA = juvenile idiopathic arthritis, SNPs = single-nucleotide polymorphisms.

### 2.2. Data sources

All datasets were obtained from publicly accessible GWAS repositories. JIA summary statistics were derived from FinnGen Release 9 and included 788 cases and 172,834 controls of European ancestry. Summary-level data for 91 circulating inflammatory proteins were obtained from a meta-analysis of 11 cohorts comprising 14,824 participants of European ancestry. Protein quantification and GWAS procedures are described in the original publications. Summary statistics were accessed through the PHPC protein resource and the European Bioinformatics Institute GWAS Catalog. The exposure and outcome datasets had no reported sample overlap.

### 2.3. Selection of instrumental variables

Single-nucleotide polymorphisms (SNPs) were selected as IVs using the following prespecified criteria:

A conventional genome-wide significance threshold of *P* < 5 × 10^−8^ was used to reduce false-positive instrument selection and weak-instrument bias. All 91 cytokines had eligible instruments after the prespecified filtering and entered the screening analysis; no relaxed association threshold was used. After harmonization and linkage disequilibrium (LD) pruning, 19, 20, and 16 SNPs instrumented C–C motif chemokine ligand 19 (CCL19), fibroblast growth factor 19 (FGF19), and monocyte chemotactic protein 3 (MCP-3), respectively.

To ensure instrument independence, LD pruning used a 10,000-kb window and an *r*^2^ threshold of 0.001.^[17]^

Palindromic SNPs or those with ambiguous allele frequencies were excluded during data harmonization.

Instrument strength was evaluated with the *F* statistic; SNPs with *F* > 10 were considered sufficiently strong.^[18]^

### 2.4. Mendelian randomization and sensitivity analyses

The random-effects inverse-variance weighted (IVW) method was the primary estimator because of its statistical efficiency when average horizontal pleiotropy is absent.^[19,20]^ MR-Egger, weighted median, simple mode, and weighted mode estimates were used to examine consistency under different validity assumptions.^[21,22]^

Cochran’s *Q* was used to assess heterogeneity in IVW and MR-Egger models.^[23]^ The MR-Egger intercept was used to assess directional horizontal pleiotropy^[21]^; Mendelian Randomization Pleiotropy RESidual Sum and Outlier (MR-PRESSO) was used to detect outlier variants and generate outlier-corrected estimates when applicable; and leave-one-out analysis was used to examine the influence of individual SNPs.^[24]^ The MR-Steiger test was used to evaluate whether the data were more consistent with an exposure-to-outcome orientation than with an outcome-to-exposure orientation.^[25]^ It was not interpreted as proof of biological directionality.

The I^2^GX statistic, estimated from the proportion of exposure variance explained and the exposure GWAS sample size (N = 14,824) using I^2^GX = (*F̄* − 1)/*F̄*, was 97.6% for CCL19, 97.3% for FGF19, and 97.4% for MCP-3. These values exceeded the 90% threshold and indicated limited dilution under the MR-Egger no measurement error assumption. Analyses were performed in R version 4.3.1 (R Foundation for Statistical Computing) using the TwoSampleMR (MRC Integrative Epidemiology Unit, University of Bristol) and MR-PRESSO (Brigham and Women’s Hospital, Harvard Medical School) packages.

### 2.5. Statistical analysis

Two-sided *P* < .05 was used to identify nominal associations in the screening analysis. To account for testing 91 cytokines, statistical significance for the primary IVW analysis required *P* < .05/91 = 5.49 × 10^−4^. Results with 5.49 × 10^−4^ ≤ *P* < .05 were classified as nominal, hypothesis-generating findings. The MR-Egger intercept and Cochran’s *Q* tests used *P* < .05 for evidence of directional pleiotropy and heterogeneity, respectively. Random-effects IVW estimates were reported in the presence of heterogeneity, and leave-one-out analysis was used to evaluate estimate stability.

## 3. Results

### 3.1. Main findings

CCL19 was instrumented by 19 independent SNPs. The primary IVW estimate indicated that higher genetically predicted CCL19 levels were associated with greater JIA susceptibility (odds ratio [OR], 2.48; 95% confidence interval [CI], 1.78–3.46; *P* = 8.93 × 10^−8^). The direction of the association was positive across all 5 MR estimators (Table 1; Figs. 2 and 3A and B). The MR-Steiger test supported the exposure-to-outcome orientation. Cochran’s *Q* indicated substantial heterogeneity (Table 2; Fig. 3C). Leave-one-out analysis identified rs94669127 as an influential contributor, but exclusion of individual SNPs did not reverse the direction of the pooled estimate (Fig. 3D).

**Table 1:** Mendelian randomization analysis of the effects of inflammatory cytokines on juvenile idiopathic arthritis.

Outcome	Exposure	Method	nSNP	β	SE	*P*	PVE (%)	OR	95% CI lower	95% CI upper
JIA	CCL19	MR-Egger	19	1.238	0.231	**5.12E−5**	5.09	3.45	2.19	5.42
JIA	CCL19	Weighted median	19	1.022	0.190	**7.48E−8**	5.09	2.78	1.92	4.03
JIA	CCL19	Inverse-variance weighted	19	0.908	0.170	**8.93E−8**	5.09	2.48	1.78	3.46
JIA	CCL19	Simple mode	19	1.006	0.396	**.020**	5.09	2.74	1.26	5.94
JIA	CCL19	Weighted mode	19	1.386	0.141	**1.11E−8**	5.09	4.00	3.04	5.27
JIA	FGF19	MR-Egger	20	−0.288	0.302	.353	4.76	0.75	0.41	1.36
JIA	FGF19	Weighted median	20	−0.222	0.167	.184	4.76	0.80	0.58	1.11
JIA	FGF19	Inverse-variance weighted	20	−0.254	0.121	**.036**	4.76	0.78	0.61	0.98
JIA	FGF19	Simple mode	20	−0.483	0.311	.137	4.76	0.62	0.34	1.13
JIA	FGF19	Weighted mode	20	−0.410	0.199	.0536	4.76	0.66	0.45	0.98
JIA	MCP-3	MR-Egger	16	−0.201	0.254	.442	4.04	0.82	0.50	1.35
JIA	MCP-3	Weighted median	16	−0.170	0.166	.306	4.04	0.84	0.61	1.17
JIA	MCP-3	Inverse-variance weighted	16	−0.241	0.117	**.039**	4.04	0.79	0.62	0.99
JIA	MCP-3	Simple mode	16	0.045	0.246	.858	4.04	1.05	0.65	1.70
JIA	MCP-3	Weighted mode	16	−0.121	0.182	.515	4.04	0.89	0.62	1.27

Bold *P* values indicate nominal significance (*P* < .05). After Bonferroni correction for 91 tests (threshold *P* < 5.49 × 10^−4^), only CCL19 remained significant in the primary IVW analysis.

β = beta coefficient, CCL19 = C–C motif chemokine ligand 19, CI = confidence interval, FGF19 = fibroblast growth factor 19, IVW = inverse-variance weighted, JIA = juvenile idiopathic arthritis, MCP-3 = monocyte chemotactic protein 3, MR = Mendelian randomization, nSNP = number of single-nucleotide polymorphism instruments, OR = odds ratio, PVE = proportion of variance explained, SE = standard error.

**Table 2:** Results of heterogeneity and pleiotropy tests in the Mendelian randomization analysis of juvenile idiopathic arthritis and cytokines.

Outcome	Exposure	Heterogeneity				Horizontal pleiotropy		
		Method	*Q*	df	*Q P* value	MR-Egger intercept	SE	*P*
JIA	CCL19	MR-Egger	69.96	17	**2.19E−8**	−0.074	0.038	.067
JIA	CCL19	Inverse-variance weighted	85.78	18	**8.18E−11**			
JIA	FGF19	MR-Egger	21.98	18	.233	0.004	0.034	.903
JIA	FGF19	Inverse-variance weighted	22.00	19	.284			
JIA	MCP-3	MR-Egger	14.42	14	.419	−0.006	0.031	.860
JIA	MCP-3	Inverse-variance weighted	14.46	15	.491			

Bold *P* values indicate *P* < .05. Heterogeneity was assessed using Cochran’s *Q* test; horizontal pleiotropy was assessed using the MR-Egger intercept.

CCL19 = C–C motif chemokine ligand 19, df = degrees of freedom, FGF19 = fibroblast growth factor 19, JIA = juvenile idiopathic arthritis, MCP-3 = monocyte chemotactic protein 3, MR = Mendelian randomization, *Q* = Cochran’s *Q* statistic, SE = standard error.

**Figure 2.**
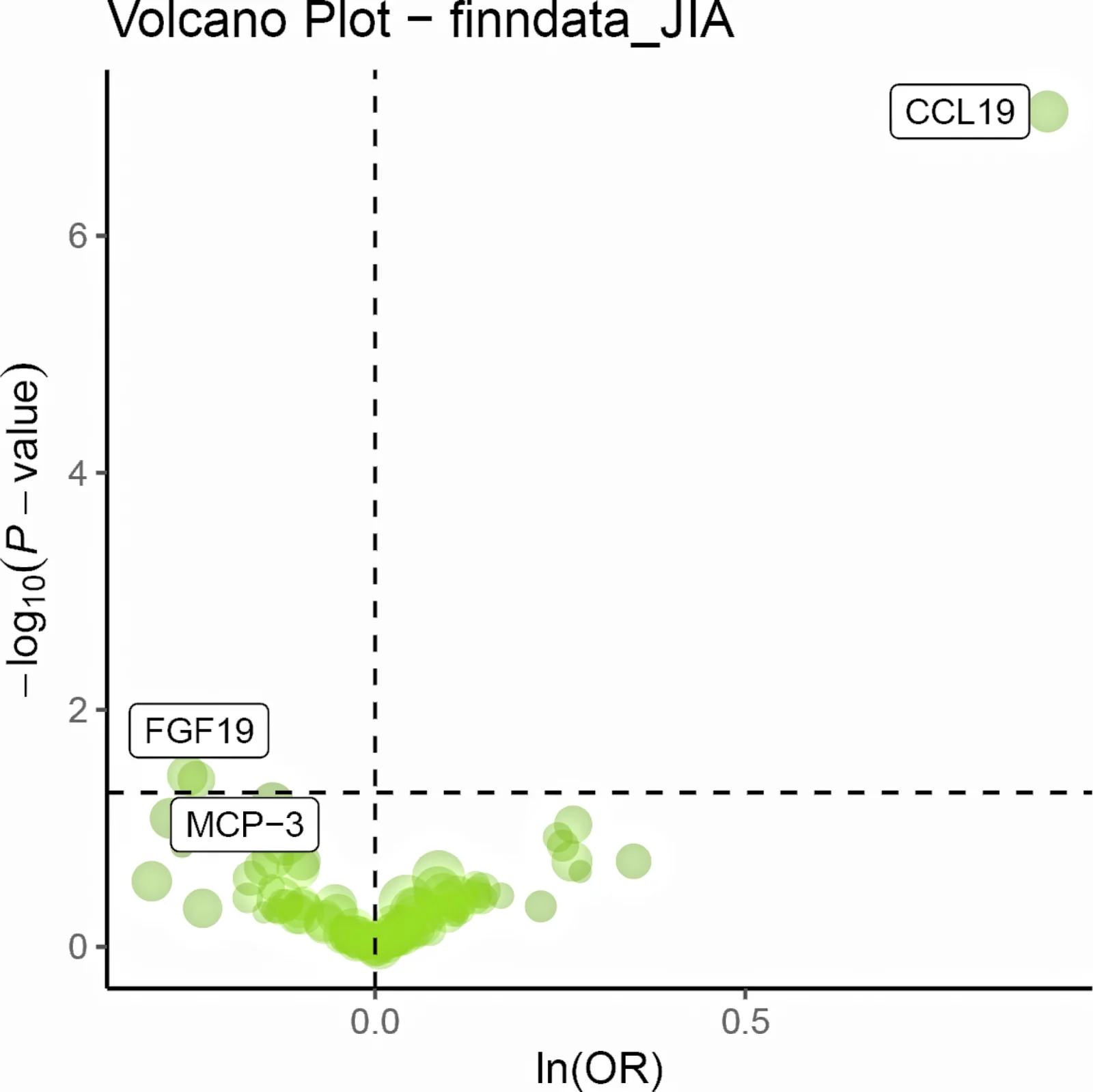
Volcano plot of inverse-variance weighted (IVW) Mendelian randomization estimates for 91 circulating inflammatory cytokines and juvenile idiopathic arthritis (JIA) susceptibility. The x-axis shows the natural logarithm of the odds ratio [ln(OR)] from the IVW analysis, and the y-axis shows −log_10_(*P*). The dashed line denotes nominal *P* = .05; only CCL19 met the Bonferroni threshold of *P* < 5.49 × 10^−4^. Positive ln(OR) values indicate that higher genetically predicted cytokine levels are associated with greater JIA susceptibility, whereas negative values indicate inverse associations. CCL19 = C–C motif chemokine ligand 19, FGF19 = fibroblast growth factor 19, MCP-3 = monocyte chemotactic protein 3, OR = odds ratio.

**Figure 3.**
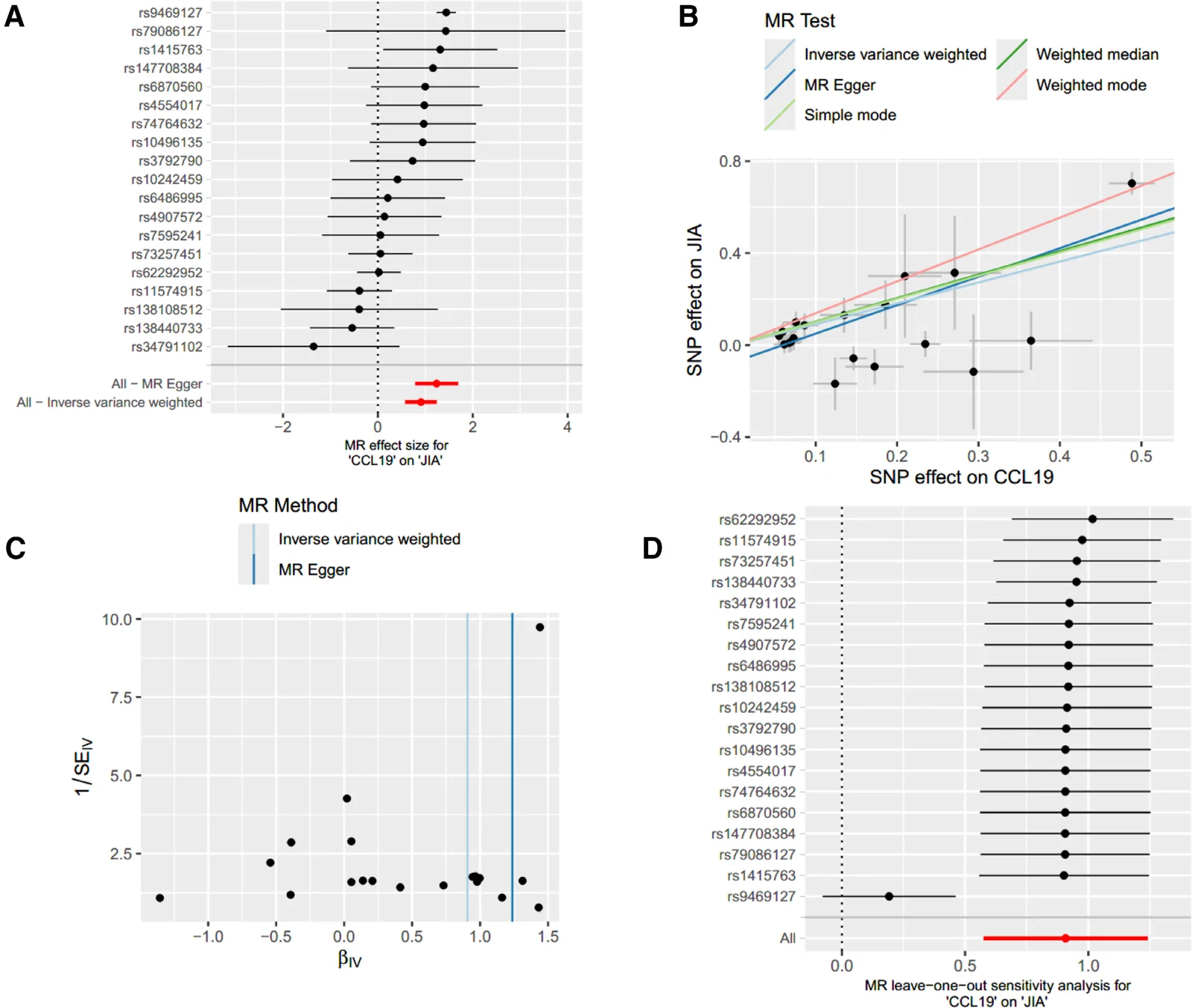
Mendelian randomization analyses of genetically predicted C–C motif chemokine ligand 19 (CCL19) levels and juvenile idiopathic arthritis (JIA) susceptibility. (A) Forest plot of single-nucleotide polymorphism (SNP)-specific and pooled estimates. Black points show the log odds ratio for each SNP instrument, and red points show pooled estimates from the 5 Mendelian randomization (MR) methods. (B) Scatter plot of SNP effects on CCL19 and JIA. Line slopes represent the estimate from each MR method. (C) Funnel plot used to assess visual symmetry and between-instrument heterogeneity. (D) Leave-one-out analysis. Points show pooled estimates obtained after sequential exclusion of each SNP instrument. IV = instrumental variable, SE = standard error.

The MR-Egger intercept did not provide statistical evidence of directional horizontal pleiotropy (intercept = −0.074, SE = 0.038; *P* = .067; Table 2). MR-PRESSO detected CCL19 outliers, and the outlier-corrected estimate was attenuated relative to the raw IVW result (β = 0.370, OR = 1.45, *P* = .041 vs β = 0.908, OR = 2.48), indicating uncertainty in the magnitude of the association. No outlier-corrected estimate was generated for FGF19 or MCP-3 (Table 3).

**Table 3:** MR-PRESSO analysis results for inflammatory cytokines and JIA.

Dataset	Exposure	Trait	MR-PRESSO estimate	Causal estimate	SD	*T* statistic	*P*
GCST90274765.1	CCL19	beta.exposure	Raw	0.908	0.170	5.35	**4.41E−5**
GCST90274765.2	CCL19	beta.exposure	Outlier-corrected	0.370	0.165	2.24	**.041**
GCST90274787.1	FGF19	beta.exposure	Raw	−0.254	0.121	−2.10	**.0495**
GCST90274787.2	FGF19	beta.exposure	Outlier-corrected	NA	NA	NA	NA
GCST90274823.1	MCP-3	beta.exposure	Raw	−0.241	0.115	−2.11	.0526
GCST90274823.2	MCP-3	beta.exposure	Outlier-corrected	NA	NA	NA	NA

Bold *P* values indicate nominal significance (*P* < .05). “Raw” denotes the estimate before outlier correction; “outlier-corrected” denotes the estimate after removal of detected outliers.

CCL19 = C–C motif chemokine ligand 19, FGF19 = fibroblast growth factor 19, JIA = juvenile idiopathic arthritis, MCP-3 = monocyte chemotactic protein 3, MR-PRESSO = Mendelian Randomization Pleiotropy RESidual Sum and Outlier, NA = not available, SD = standard deviation.

At the nominal *P* < .05 threshold, IVW estimates suggested inverse associations for FGF19 (OR, 0.78; 95% CI, 0.61–0.98; *P* = .036) and MCP-3 (OR, 0.79; 95% CI, 0.62–0.99; *P* = .039). Neither association met the Bonferroni threshold of *P* < 5.49 × 10^−4^. CCL19 was the only cytokine that remained significant after correction (unadjusted IVW *P* = 8.93 × 10^−8^; Bonferroni-adjusted *P* = 8.12 × 10^−6^); therefore, the nominal FGF19 and MCP-3 results were treated as hypothesis-generating findings rather than confirmatory evidence.

## 4. Discussion

In this two-sample MR analysis of 91 circulating inflammatory cytokines, higher genetically predicted CCL19 levels were associated with greater JIA susceptibility among 788 cases and 172,834 controls of European ancestry in FinnGen. CCL19 was the only cytokine that met the Bonferroni-corrected threshold. The nominal inverse estimates for FGF19 and MCP-3 did not survive multiple-testing correction and should not be interpreted as established protective effects.

The CCL19 result prioritizes an association for further study but does not establish CCL19 as a pathogenic mediator, diagnostic biomarker, or therapeutic target. MR estimates reflect differences in lifelong genetically proxied circulating levels under specific instrument assumptions. They do not demonstrate that short-term pharmacological modulation will alter disease risk, and they do not replace experimental or clinical validation.

Biological evidence nevertheless provides a plausible context. Through C–C chemokine receptor 7 (CCR7), CCL19 regulates dendritic-cell and T-cell trafficking, lymphoid organization, and adaptive immune responses.^[26,27]^ Dysregulated CCL19–CCR7 signaling could, therefore, influence immune-cell recruitment to inflamed tissue.^[28–30]^ The present genetic association is compatible with this pathway, but it does not identify the relevant tissue, cell type, disease stage, or molecular mechanism in JIA.

The absence of Bonferroni-supported associations for classical inflammatory cytokines such as IL-6 or TNF-α does not contradict their established roles in active JIA. MR captures genetically proxied, generally lifelong circulating concentrations, whereas acute or local synovial signaling, developmental timing, and treatment-responsive pathways may not be well represented. Differences across cytokine GWASs in the numbers and strengths of instruments, variance explained, and measurement precision also affect statistical power. CCL19 may, therefore, have emerged because its genetic instruments captured a detectable component of susceptibility, not because it is uniquely more important than IL-6 or TNF-α.

The CCL19 estimate requires caution because heterogeneity was substantial in both IVW (*Q* = 85.78, df = 18; *P* = 8.18 × 10^−11^) and MR-Egger (*Q* = 69.96, df = 17; *P* = 2.19 × 10^−8^) analyses. Although the MR-Egger intercept was not significant, that test cannot exclude all forms of horizontal pleiotropy. The MR-PRESSO estimate was also markedly attenuated after outlier correction. Thus, the direction of the genetically predicted association was positive across analyses, but the raw IVW estimate may overstate its magnitude.

FGF19 regulates metabolic signaling and has reported antioxidant and antifibrotic effects,^[31–37]^ whereas MCP-3/CCL7 recruits monocytes and other immune cells to inflammatory sites.^[38–40]^ These functions make both cytokines biologically relevant, but their nominal MR estimates were not robust to correction and may represent chance findings. These hypothesis-generating findings require evaluation in better-powered datasets.

A major limitation is the clinical heterogeneity of JIA. The FinnGen outcome combined JIA cases into a single phenotype, whereas International League of Associations for Rheumatology categories – including systemic, oligoarticular, polyarticular, and enthesitis-related disease – have distinct immunopathological features. Pooling these categories may dilute subtype-specific associations or produce an average estimate that does not apply to any single subtype. Accordingly, the present analysis cannot support subtype-specific inference.

The outcome GWAS included only 788 cases. This small number of cases reduces power to detect modest associations, increases uncertainty in MR estimates, and raises the likelihood of false-negative results; it may partly explain the failure of FGF19, MCP-3, and other cytokines to meet the corrected threshold. In addition, all exposure and outcome data were obtained from participants of European ancestry. Differences in allele frequency, LD, cytokine regulation, and autoimmune disease architecture may limit transferability to Asian, African, and other populations. Moreover, unmeasured environmental and clinical factors that may influence both circulating cytokine concentrations and JIA susceptibility, including childhood infections, socioeconomic status, and prior or ongoing anti-inflammatory treatment, cannot be excluded and could contribute to residual confounding. Finally, residual horizontal pleiotropy, nonlinear effects, developmental timing, and tissue-specific cytokine action cannot be fully evaluated with the available summary statistics.

Future studies should replicate the genetically predicted CCL19 association in larger, independent, and ancestrally diverse JIA GWAS cohorts with subtype-resolved phenotypes. Multivariable MR could assess correlated cytokine pathways, colocalization could test whether cytokine and JIA associations share causal variants, and functional laboratory experiments could determine whether CCL19–CCR7 signaling alters disease-relevant immune-cell behavior. Prospective clinical studies would then be required to evaluate biomarker or therapeutic relevance.

In conclusion, genetically predicted higher CCL19 levels were associated with greater JIA susceptibility in participants of European ancestry, whereas the FGF19 and MCP-3 results were nominal and hypothesis-generating. Given outcome heterogeneity, the small number of cases, and attenuation after outlier correction, the CCL19 result should also be considered a hypothesis-generating finding, not evidence of a causal or clinically predictive relationship. Independent genetic, functional, and clinical validation is required before any diagnostic or therapeutic inference.

## Author contributions

**Conceptualization:** Pingping Liu, Wanyue Qin, Yifei Feng.

**Data curation:** Jian Pang, Pingping Liu, Yifei Feng.

**Formal analysis:** Jinhua Yang.

**Methodology:** Wanyue Qin, Jinhua Yang, Yifei Feng.

**Resources:** Lijia Pan.

**Software:** Jian Pang, Lijia Pan, Pingping Liu.

**Supervision:** Sheng He, Shuihua Yang.

**Visualization:** Lijia Pan, Wanyue Qin, Jinhua Yang.

**Writing – original draft:** Jian Pang.

**Writing – review & editing:** Shuihua Yang.
